# Bflinks: Reliable Bugfix Links via Bidirectional References and Tuned Heuristics

**DOI:** 10.1155/2014/701357

**Published:** 2014-10-29

**Authors:** Lutz Prechelt, Alexander Pepper

**Affiliations:** ^1^Institut für Informatik, Freie Universität Berlin, 14195 Berlin, Germany; ^2^Infopark AG, Kitzingstraße 15, 12277 Berlin, Germany

## Abstract

*Background.* Data from software version archives and defect databases can be used for defect insertion circumstance analysis and defect prediction. The first step in such analyses is identifying defect-correcting changes in the version archive (bugfix commits) and enriching them with additional metadata by establishing bugfix links to corresponding entries in the defect database. Candidate bugfix commits are typically identified via heuristic string matching on the commit message. *Research Questions.* Which filters could be used to obtain a set of bugfix links? How to tune their parameters? What accuracy is achieved? *Method.* We analyze a modular set of seven independent filters, including new ones that make use of reverse links, and evaluate visual heuristics for setting cutoff parameters. For a commercial repository, a product expert manually verifies over 2500 links to validate the results with unprecedented accuracy. *Results.* The heuristics pick a very good parameter value for five filters and a reasonably good one for the sixth. The combined filtering, called bflinks, provides 93% precision and only 7% results loss. *Conclusion.* Bflinks can provide high-quality results and adapts to repositories with different properties.

## 1. Introduction

The five-level CMMI software process maturity framework [1] suggests that software processes can be managed towards higher performance when driven by software process measurement data (level 4) and that iterative process optimization needs to rely on such data (level 5). Unfortunately, obtaining process data can be costly unless a high degree of automation is achieved in collecting and validating it.

Some researchers suggested that we should study how to systematically exploit* existing* data stored in software repositories that we create and maintain anyway, such as source code version archives, configuration management databases, requirements management databases, defect tracking databases, and possibly even less structured ones such as wikis and mailing lists. Since 2004 this idea has sprouted into a regular series of initially workshops and now conferences under the name of Mining Software Repositories (MSR, http://www.msrconf.org); it has produced a wealth of interesting ideas and approaches.

One of the most potentially useful application areas when mining software repository data is understanding defect insertion and defect removal processes [2]. Consequently, a large number of MSR type works revolve around defects (*bugs*) in one way or another. The main repositories of interest for such works are commonly the source code version repository (or* version archive* for short, for example, using CVS, Subversion, or Git) and the defect and defect correction history description database (or* bugtracker* for short, e.g., using Bugzilla).

Unfortunately, it is quite difficult to identify defect insertions. The common first step in defect-related MSR analyses is to identify transactions in the version archive that correct a defect, usually called* bugfix commits*. In the simplest approach, this is done by scanning the check-in comments (*commit messages*) for words such as “bug” and “fixed” [3], which is a somewhat unreliable method even with optimal selection terms. An improved and extended approach thus attempts to connect a bugfix commit to a matching entry or entries in the bugtracker that describe the bug being fixed [4] and whose number is mentioned in the commit message. If successful, this also makes much additional data available from the bug entry to be used for whatever bug-related analysis is intended afterwards (such as time-to-detect, time-to-fix, and severity classes).

We call such a pair of bugfix commit and bugtracker entry a* bugfix link*. Our goal is, given a repository, to find as many correct (i.e., valid) bugfix links as possible while avoiding false positives (i.e., invalid ones). We call our method* bflinks*; the approach is first* generating* a set of* candidate bugfix links* and then using a chain of* filters* to weed out most of the false ones.

For the present study, we define a* bugfix* to be the content of any commit to the version archive whose purpose is primarily to address the issue described in a specific entry in the bugtracker, no matter whether that describes a defect or some other type of change request.

The study describes how practicing engineers at a company, Infopark, went to establish bugfix links in order to (later on) perform actual bugfix data analysis for engineering purposes. This is in contrast to researchers performing such analysis for research purposes and has a number of impacts on the contents and noncontents of the paper.

We will proceed to describe the design rationale of our study and its research contributions (Section 2), the research context at Infopark and how Infopark's data repository was created (Section 3), our overall approach and its related work (Section 4), the specific bugfix link identification methods investigated (Section 5), the results thus obtained (Section 6), and the procedure for applying the overall method to other repositories in practice (Section 7). We finally discuss threats to validity (Section 8) and present our conclusions in Section 9.

## 2. Design Rationale and Research Contributions

This section describes why we set up the study the way we did and what scientific (certainty-oriented) and engineering (cost-/benefit-oriented) contributions we claim it to make.

(1) Previous studies on linking commits to issue reports have mostly considered open source software systems only (as opposed to commercial closed source). Most of the few uses of closed source data in MSR works are either vague with respect to the origin and nature of the data (e.g., [5] or [6]) or do not contain specifics such as concrete defect counts and so on (e.g., [7]). Where the same MSR technique has been evaluated for both open source repositories and commercial repositories, quite different results have sometimes been found, for example, by [8] versus [6]. For bugfix link determination, it has not yet been considered that closed source development processes are different from open source ones and the resulting repositories might thus have different characteristics that could perhaps be exploited for finding bugfix links. In the present work, we therefore consider the following to be a scientific contribution. We present results from a closed source SVN and Git version repository; we describe both the product and the process from which it originates and also include concrete numbers (Section 3); we analyze the repository in depth and look for ways to extract bugfix links that differ from the ways previously used for open source data. This contribution represents a tradeoff against the possibility of publishing the raw data (for full reproducibility) as it can be done for open source data.

(2) We do indeed find a characteristic in our data that has not previously been used: explicit reverse links contained in a bugtracker entry that point to a commit. To exploit it, we introduce new filtering heuristics (Sections 5.4, 5.6, and 5.8), one of which turns out to greatly improve the overall result (Section 6.9). We consider this as an engineering contribution.

(3) We describe and analyze the filters individually so they can be used in a modular fashion and users can understand for what types of repository it might be sensible to use or not use certain filters (Section 6). Compared to previous works that lump several generation and filtering heuristics into one single monolithic step, this is a scientific and engineering contribution.

(4) For some bugfix link candidates, it is difficult to understand whether they are valid or not. We aim at being always correct in this judgment by employing a company insider product expert who manually checked more than 2500 bugfix link candidates carefully (Section 4.5). This is important because some of the filters affect only small numbers of bugfix link candidates and so even a handful of overlooked or additional bugfix links could distort these filters' quality assessment results massively. The wording with which previous works describe their validation effort often points to much less thorough procedures. For example, [5] says “[...] manual inspection for false positives e.g., [sic!], whether there are identified bug report numbers which cannot be truly a bug report link.” and [4] even performs no overall manual validation at all: “Based on a manual inspection of several randomly chosen links [...], we decided to use only those links whose syntactic and semantic levels of confidence satisfy the following condition: [...].” We therefore consider the unprecedented accuracy of our results to be a scientific contribution. This contribution represents a tradeoff against the possibility of assessing the bugfix finding method using a broader set of multiple repositories.

(5) Most filters involve a cutoff threshold or similar settable parameter. Successful use of the filtering chain requires adjusting these parameters to the characteristics of the given repository, because unsuitable settings can utterly ruin the filter chain's performance. To our knowledge, our work is the first to provide guidance for this step; we describe metaheuristics for setting the filtering heuristics' cutoff parameters in Sections 6.3 to 6.7. As we know of no other work that describes how to select parameter values (most works do not even assure the reader that the values used have been obtained without looking at the resulting performance, which means they may involve overfitting and thus exaggerate the true performance of the method), we consider this as an engineering contribution.

(6) While a scientific study of bugfix link identification will perform extensive manual validation of the results, a practicing engineer can at best afford manual validation for a small sample. We therefore sketch the overall procedure of how one would apply bflinks in practice without a comprehensive manual validation and discuss how to adapt to different properties of the repository at hand (Section 7). We consider this as our main (engineering) contribution and the others being sort of an infrastructure for it.

Summing up, our contributions focus on achieving practical applicability of the technique while ensuring high validity of the results. In contrast, it is expressedly* not* a goal to improve the recognition performance beyond that of other works, because such performance greatly depends on the particular dataset and as explained under (4) above we will not use a multitude of datasets.

On a methodological level, we explain why absolute measurements of recall are problematic (Section 4.3) and propose the use of relative measures (*results loss*, in our case) as a replacement (Section 4.4).

## 3. Research Context and Data

### 3.1. Context and Quality Criteria

This work was started by a company, Infopark AG, with the intention of achieving insights regarding their own defect insertion and removal processes that could be turned into process improvements.

It was planned to (1) identify bugfix commits, (2) establish their bugfix links, (3) identify the corresponding bug commits (defect insertions), and (4) analyze them for interesting patterns.

Steps 3 and 4 turned out to be infeasible [9], so this work reports on steps 1 and 2 only.

Infopark identified* incorrect conclusions* as a major risk in this work, as those might lead to fruitless process change effort or even counterproductive process changes. Therefore, Infopark required that the analysis methods should be chosen such that their recall (the fraction of all valid objects of the overall set that appear in the subset that was found) would be about 50% or higher (to avoid irrepresentativeness) and their precision (the fraction of all objects in the subset found that are valid) would be at least 80% or preferably 90% (to avoid distortion from misleading false positives). Such data quality requirements are sensible because it is known that the results at least of defect prediction analysis are sensitive to biases in their input data [10] and that the completeness of bugfix links tends to be low [10, 11], which makes bias likely.

We perform the study on only Infopark's repository rather than several because accurate assessment of precision requires the correct manual classification of many commits—which requires background knowledge of the respective product and its development practices.

### 3.2. Company, Domain, Technology, and Development Style

Infopark is an early Web company. Founded in 1994, it built the first version of its main product CMS Fiona in 1997. CMS Fiona is a content management system (CMS) aiming at large-scale and high-traffic web sites with both static and dynamic parts. Its particular strengths lie in consistency-keeping for static content. Two other products represented in our repository data, the Online Marketing Cockpit and an internal product, are so much smaller that they hardly influence the results of our investigation at all.

Infopark has always been very open for innovation not only in its products but also in the technologies and processes it used for building them. Therefore, the data investigated represents, over time, a multitude of both technical platforms for managing it and programming languages used. CMS Fiona was originally started in Objective *C*, then extended in Tcl, then largely transformed into Java, and is recently being extended and partially reimplemented in Ruby for central hosting in the cloud.

The following properties of Infopark will be relevant for the current study.

- In the CMS domain, feature requests are very frequent and there is no clear line between avoidable bugs and feature requests. Consequently, Infopark often treats the implementation of small improvements to the functionality just like bugs and such improvements represent a substantial fraction of our “bugfix” data. Because feature requests typically involve more code than ordinary bugfixes, our data contains many nonsmall bugfixes.
- Infopark has always had low turnover of staff and was therefore able to follow intended processes and good practices stably, in particular (iii) and (iv).
- The commit message of a bugfix commit typically mentions the number of the corresponding bugtracker entry.
- When closing a bugtracker entry, a comment will often be added that mentions the version number of the corresponding bugfix commit.

### 3.3. History of the Version and Bug History

The earliest version archive data that is available for our analysis starts in the year 2000 and takes the form of a* Concurrent Version System* (CVS) archive. In late 2003, Infopark switched to* Subversion* (SVN) as the version archive software and in 2008 it switched to* Git*. In both transitions, the new archive started with a snapshot of the old archive's main branches; the previous revision histories were not migrated, but the old archives have been preserved.

The only bug tracking data that is still available (and extends until the present) comes from an instance of* Bugzilla* which contains one single continuous bug history; it was introduced in early 2003 and so matches only a fraction of the CVS version archive (which eventually we did not use, see Section 5.2) but all of the SVN and Git archives.

### 3.4. Data Preparation and Data Size

For the analysis presented here, we created one single continuous Git archive that covers all commits (including branches) from the Infopark CVS and SVN and Git archives which contain 25 653, 14 694, and 5 261 commits, respectively; more than 45 000 commits overall, created over roughly 11 years of development. We added artificial bridging commits leading into each initial state of each subsequent subrepository; we kept all original version identifiers (and in fact all relevant metadata used in our analysis) in a separate database as extracted by the MininGit (https://github.com/SoftwareIntrospectionLab/MininGit) software (a fork of CVSAnalY [12]), to which we contributed a number of improvements during the debugging phase of the extraction process.

Since we are interested in bugfixes and those are defined to relate to bugtracker entries, we use only those commits leading towards the product releases made since the introduction of Bugzilla. These data consist of 31 854 commits which represent a total of 263 033 file-level deltas comprising 21 995 065 line-changes altogether. Only 2% of the commits are lacking the commit message.

The Bugzilla database contains 9 444 bug entries with a total of 46 302 comments. 60% of the entries are marked as repaired, 9% as duplicates, and 24% as invalid or “works for me.” According to results reported in [13], these are ordinary values.

## 4. Overall Approach

Our approach for establishing a set of bugfix links consists of two methods for generating* candidate* bugfix links (high recall and low precision) and several filters for rejecting some of those candidates in order to improve precision at a hopefully small loss of recall.

### 4.1. Generators and Filters

The two generator methods are as follows.

- *BM:* searching for IDs of bugtracker entries mentioned in commit messages, and
- *CM:* searching for IDs of commits mentioned in bugtracker entries.

The filters are as follows.

- *FB* (frequent Bugzilla IDs): reject all candidates that involve a Bugzilla ID which occurs in many other commit messages as well.
- *FC* (frequent commit IDs): reject all candidates that involve a commit ID which occurs in many other Bugzilla entries as well.
- *SB* (small Bugzilla IDs): reject all candidate Bugzilla IDs that are small.
- *TT* (time travel): reject all candidates that violate causality by having a referencing point that was written before the referenced point existed.
- *LU* (late update): reject all candidates where the bugzilla update (if any) occurs an overly long time after the corresponding commit.
- *UD* (unidirectional): reject all candidates where only one direction of bugfix link reference is available (either from Bugzilla to commit or from commit to Bugzilla, but not both).

If it appears appropriate for a given dataset, any one of the filters could be left out, so the approach is customizable. Before all of these, the filter* NX*, “reject all candidates whose commit ID or Bugzilla ID does not exist,” is implicitly applied as a trivial and flawless first filtering stage.

### 4.2. Related Work

Early works such as [3] that mined bugfix data involved a single repository only, the version archive, and so would work by means of string matching on commit messages for terms such as “bug,” “patch,” and “fix” only. Even recent research such as [14] sometimes takes this approach if details about the nature of the defect are not of interest and thus no bugfix links need to be formed.

Since it was introduced by [15], more advanced approaches connect the version archive information to the bugtracker information and so involve bugfix links in the sense used here and hence possibly also some filters such as NX, FB, SB, TT, LU, or similar ideas, for example, in [10, 13, 16–19].

The present paper appears to be the first report involving backward links and the corresponding filters FC and UD.

ReLink [20] extends a simple search for explicit links (based on refined string pattern search, filter NX, and “fixed” status of the bugtracker entries) with a search for implicit ones. It radically considers* every* pair of a commit and a bugtracker entry to be a candidate bugfix link and then uses three filters to cut these down to sensible ones as follows:

- first, a combination of TT and LU;
- second, the requirement that the author of the commit must also be a (co)author of the bugtracker entry;
- third, sufficient textual similarity (TS) between the commit message and the description and comments in the bugtracker entry. For the latter, ReLink employs stemming, stop-word elimination, and thesaurus-based word unification to reduce the vocabulary and then applies the cosine text similarity metric. The idea is that if no bug ID is provided in the commit message, it will instead talk about topics (such as failure symptoms or class or method identifiers) that are also mentioned somewhere in the (typically much longer) bugtracker entry.

MLink [21] goes still one step farther by also taking into account similarity of source code fragments and other text mentioned in the tracker entry with the actual commit source code content.

MLink and ReLink are very clever ideas, which could nicely be combined with the ideas of bflinks. When a dataset has a great deal of explicit bugfix links (as the Infopark data does) bflinks will work well, while for other datasets ReLink and MLink can improve the otherwise low recall with hopefully sufficient precision.

Note, however, that ReLink's LU and TS both require cutoff parameters and their values are extremely critical for good precision because of the huge number of incorrect candidate links that need to be rejected.

This is not a small issue. For instance, the smallest of the three datasets investigated, ZXing, has 1 694 commits and 135 fixed bugs, hence, over 220 000 candidate bugfix links. The reported precision is 91% (107 of 118 correct), but if less appropriate parameter settings would result in only an additional 1% of the incorrect candidates getting through, precision would drop to 4.6% (107 of 2318 correct). For the largest of the datasets, Apache, the equivalent drop in precision, would go from 75% to 0.016% (only 0.06% of the commits from the Apache dataset, the 493-commit Linkster subset, are actually used in the paper; its Table 3 is misleading in this respect, consult the original paper [11]).

Unfortunately, in the published form of ReLink just this critical parameter setting is unsound. The published algorithm involves a fine-grained search of the parameter space. The best parameter values are identified by measuring the resulting precision and recall via a ground truth dataset (golden set). This procedure is impossible in a real application, because ground truth will not be known—if it was, no procedure for establishing bugfix links would be needed at all. Therefore, the published performance figures are based on near-optimal parameter settings that could not be achieved by a real user and are hence overoptimistic. This means that the ReLink procedure as published is utterly incomplete and cannot be applied in practice. Until a sound method for tuning the ReLink cutoff parameters is devised, we can therefore perform neither a combination nor a comparison of bflinks and ReLink.

MLink shares a similar problem, if less strongly. The authors present in detail what they call an “unsupervised hill-climbing algorithm” (which happens to be neither unsupervised nor doing hill-climbing) requiring ground truth for setting the threshold parameters but do not state what data they fed it. At least they use just one fixed set of parameters for all of their four benchmark datasets afterwards.

### 4.3. The Issue of Measuring Recall

Bugfix link identification is a form of information retrieval. From among all conceivable links, find all correct ones.

If the output of such a search is not too large, measuring its precision is practical. Manually assess each output and classify it as correct or incorrect.

Measuring recall, however, is generally difficult because it involves assessing all conceivable links. Unless this base set is rather small, complete manual assessment (and hence reliable determination of recall) is not feasible.

For instance determining the ground truth for the smallest dataset in the ReLink article involves checking 220000 pairs. Even at an unlikely fast speed of 15 seconds per pair, true individual checking of each pair would take half a person-year of effort. It appears unlikely that the authors have invested this much effort for determining their “golden set” (and then another). Instead, they will have performed a reduced, heuristic checking, which opens possibilities for overlooking correct bugfix links—and at a determined bugfix link density of only 0.065% (143 links) the fraction of overlooked ones can easily be quite large. For the Infopark dataset, establishing a complete ground truth would even involve checking over 300 million pairs (or 700 person years at 15 seconds apiece).

So alternative approaches to complete manual checking have to be devised in a domain-dependent manner. See, for instance, the ingenious approach taken by the TREC text retrieval contest [22] that exploits the fact that many different answers (from different retrieval systems) are available for the same set of queries.

In the bugfix link identification domain, we see four approaches that have been used to obtain estimates of recall.

1. Heuristic manual checking aimed at a good approximation of the complete ground truth for small repositories (ZXing and OpenIntent of ReLink). This approach will be inaccurate if too many links are overlooked.
2. Tool-supported manual checking by a project expert aimed at a good approximation of a partial ground truth for a large repository (Linkster/Apache of ReLink). This case stems from [11] and covers 6 weeks (493 commits) of the Apache project only. This approach will be inaccurate if too many links are overlooked or the chosen time period is not representative for the project overall. Partial checking of a* random* sample would be much harder for the expert because of the loss of context-carryover due to the nonconsecutiveness of the commits.
3. A survey aiming at a rough estimate of overall recall for a very large repository. This was used at Infopark and will be described in Section 4.5 and evaluated in Section 6.2. The survey approach is inherently imprecise and likely inaccurate. It serves as a low-cost way to obtain a ballpark figure for recall from existing relative measures.
4. Relative measures similar to recall (results gain for augmentation methods such as ReLink and results loss for filtering methods such as bflinks). This approach avoids the need for a ground truth completely and instead requires only the checking of links proposed (perhaps at some internal stage) by the respective system. It will be described in the next subsection.

It is feasible to avoid relying on ground truth datasets for bugfix link identification. For research purposes, relative measures are often sufficient for comparing different systems and much cheaper than establishing ground truth. For practical engineering purposes, the survey method is sufficient for classifying recall as good enough or not good enough and again cheaper than establishing ground truth.

We go on to describe the measures we use in the present study to characterize the quality of the individual bflinks filters.

### 4.4. Measurement Metrics

After the generators, the filters can be used individually or in arbitrary combination. Assume that we have as input *N* generated candidate bugfix links of which *C* are correct and *W* are wrong. *N* = *C* + *W*. After filtering, we are left with an output of *n* proposed bugfix links, of which *c* are correct and *w* are wrong. *n* = *c* + *w*. For a certain combination of generators and filters we speak of overall precision (*op*) as our main quality metric. What fraction of the proposed bugfix links represents true, valid bugfix links? *op* = *c*/*n*. We would also like to know overall recall (*or*): What fraction of all true, valid bugfix links are actually returned after filtering? *or* = *c*/*C*. Determining *C* involves checking all *N* candidates and is hence extremely laborious (It is actually even worse as a fully meaningful *or* result needs to be based on *C*′, not *C*, which is even more thoroughly impractical. *C*′ is the conceptual number of* all* correct bugfix links, including even those not proposed as candidates by the generators, e.g., because no hint to the bugtracker entry is included in the commit message). Therefore, *or* is usually only approximated.

For a given filter or filtering chain, we also speak of the following.

*(i) Filtering Precision (fp).* What fractions of the items rejected by the filter are indeed false positive candidates as intended? Do not let the term confuse you; overall precision talks about the output, but filtering precision talks about what does* not* appear in the output: *fp* = (*W* − *w*)/(*N* − *n*). 100% filtering precision means no correct candidate is rejected. A filter will always improve overall precision if its filtering precision is better than 50% and lower-precision filters can improve overall precision if overall precision is already high. 

*(ii) Filter Strength (st).* What fraction of all candidates is rejected by the filter? *st* = (*N* − *n*)/*N*. A filter may have high precision itself, but still little positive impact overall because of low strength.

*(iii) Results Loss (rl).* What fraction of all candidate links is filtered out that was valid links? This is just a combined effect of filtering precision and filter strength but shown separately for clarity. It is independent of the overall recall and thus more practical to determine but makes a statement of a similar kind. For example, a filter with strength 50% and filtering precision 70% will (by means of the 30% falsely rejected candidates) produce a results loss of 15%. Generally, *rl* = *st* · (1 − *fp*).

The next subsection describes how these metrics were operationalized.

### 4.5. Measurement Method

For measuring any of the above metrics we need to know which candidates are actually valid ones. This can only be determined by applying human judgment and background knowledge, so that manual assessment is required. For this study, we have manually checked over 2500 (52%) of our candidate bugfix links. More precisely, a long-term member of the Infopark development team has individually looked at these pairs of defect database entry and commit message and consulted the source code diff and/or colleagues where necessary in order to make a reliable decision whether a pair forms a true bugfix link or not.

For our research goal of* understanding* the precision achieved, these 52% would ideally have been a random sample. However, Infopark pursued an engineering goal:* maximize* precision without sacrificing too much recall. For this purpose, the checking exclusively covered items to be possibly rejected by the filters, so as to make the filters sufficiently strong, but not too strong. A certain parameter value was picked early on (before the development of the parameter tuning heuristics) and all candidates that would be filtered out with this parameter value were manually validated. This produced 100% checking coverage for most filter/parameter variants reported in this paper but somewhat less for some of them. Due to the candidates with validity status “unknown” (which are removed from the computations), the algebraic relationships that conceptually hold between the various statistics are only approximately correct. Filter UD, as introduced in Section 5.8, is special: here we checked the* accepted* items rather than the rejected ones, for reasons described in Section 6.9.

Unless the filters are idiotic, these candidates will contain a higher density of invalid bugfix links than the rest of the population and our sample is thus biased. The overall precision assessment from this biased sample is pessimistic and the resulting estimates of overall precision *op* (unless they are very high (>95%), so that stochastic error could play a role) will be lower than the correct ones. Since we check* all* of the possibly rejected candidates, the measurement of filtering precision *fp* is fully accurate.

No such “will be pessimistic” argument is available for overall recall *or*. So in order to avoid estimates whose bias is unknown, we rely on results loss *rl* instead which only relies on accurate measurements and is therefore also accurate.

For obtaining at least a very rough estimate of recall in order to check whether Infopark's 50% recall requirement is fulfilled, we performed a quick, informal survey of four long-time Infopark developers, asking (a) what percentage of all commits are primarily bugfix commits? and (b) what percentage of those has a corresponding Bugzilla entry? We compare the outcome to the number of bugfix links we found.

## 5. Bugfix Link Identification Method

This section explains the generator methods, filters, and tuning parameters in detail.

### 5.1. Generator BM: Bugtracker IDs Mentioned in Commit Messages

Infopark was interested in complete bugfix links only, not in solo bugfix commits without a corresponding link to a bugtracker entry. We therefore made the following early (and quite radical) decision with respect to our analysis of the commit messages. We would not perform any keyword string matching on the commit messages (for terms such as “bug,” “defect,” and “fix”) at all. Instead, we would only look for integer numbers that represented existing Bugzilla entry IDs, assume that this number constitutes a bugfix link, and then validate the correctness of that link as best we can. (Most entries are not just integers such as “1234” but rather strings such as “#1234” or “BZ1234” or similar forms. However, there was no fixed rule in place at Infopark in this regard and so we decided to go for all integers (as also suggested by [5]) to maximize recall and repair the precision penalties by filtering. This worked well).

Requiring some keyword matching in addition would improve the precision of the results at the expense of lower recall. It is quite plausible that careful application of keyword matching could improve our results somewhat, but we did not investigate this in the present study and rather present the results of a “pure” bugfix link search instead.

### 5.2. Generator CM: Commit IDs Mentioned in Bugtracker Entries

This numbers-based search approach and the disciplined Infopark development culture suggest a second data source for bugfix links that we have not yet seen analyzed in the literature yet. If commit messages might contain numbers referring to bugtracker entries, why not also look in bugtracker entry comments for numbers referencing commits (version numbers). We use all strings that look like the IDs of an existing version as candidate bugfix links as well. These IDs look very different for each versioning system. For CVS they are either single-dotted numbers (on the trunk, e.g., 1.23) or multi-dotted numbers (on a branch, e.g., 1.23.1.7); for SVN they are integers, at Infopark preceded by “r” (e.g., r1722; we accept “R” as well); for Git they are 40-digit sedecimal strings (e.g., 6050732e725c68b83c35c873ff8808dff1c406e2).

SVN and Git version IDs are unique for the whole repository and so are easy to resolve. CVS version IDs, however, are local per file, so resolving them requires finding the corresponding filename and perhaps (where filenames are not unique themselves) even pathname. This is much more difficult, hence quite error-prone, and often even impossible. Since we have only a few months worth of CVS data, we decided not to bother and leave the CVS part of the history out of our analysis entirely.

The union of these two datasets of candidate bugfix links (from commit messages and from bugtracker entry comments) form the basis for the subsequent filtering steps. Such filtering is very important: Bugzilla IDs in particular will be polluted with plenty of false positives from port numbers, RFC numbers, percentage numbers, time measurement numbers, and many other kinds. The version IDs, although far less confusable, will also have false positives, because they may be mentioned in other roles than the bugfix link role (e.g., defect was validated to be still present in r123). Our study will investigate the following filtering criteria.

### 5.3. Filter FB: Reject Overly Frequent Bugzilla IDs

An integer number found in a commit message that is really the ID of a Bugzilla entry will occur only in one or possibly a few different commit messages (for difficult bugs, needing multiple complementary fixes or fixes of fixes), but not in many.

*Heuristic FB.* Consider all bugfix links established by a Bugzilla ID *i* in a commit message to be invalid if that Bugzilla ID *i* appears in *f*_*B*_ or more different commit messages. Choose a suitable cutoff frequency *f*_*B*_.

### 5.4. Filter FC: Reject Overly Frequent Commit IDs

Even more dubious is the same commit ID appearing in multiple different Bugzilla entries because Infopark does not often check in multiple bugfixes in a single commit. Therefore, multiply appearing commit IDs probably indicate something other than a bugfix (such as an alpha release).

*Heuristic FC*. Consider all bugfix links established by a commit ID *i* in a Bugzilla entry to be invalid if that commit ID *i* appears in *f*_*C*_ or more different Bugzilla entries. Choose a suitable cutoff frequency *f*_*C*_.

### 5.5. Filter SB: Reject Small Bugzilla IDs

Small integers found in commit messages will very often not be Bugzilla IDs at all but rather other things such as percentages, HTTP status codes, buffer sizes, and item numbers. Since such numbers tend to occur repeatedly with the same value, rejecting them should improve precision. It will also not hurt recall much, because only few* proper* Bugzilla IDs are mentioned more than once.

*Heuristic SB.* Reject all candidate Bugzilla IDs smaller than a minimum *m*_*B*_. Choose a suitable minimum.

### 5.6. Filter TT: Reject Major Time Travel

It makes no sense to assume that a commit *C* made at time *t*(*C*) references a bugtracker entry *B* that was created only at a* later* time *t*_*c*_(*B*), because the author of the commit message would not be able to reliably predict the ID of that later entry; such a bugfix link violates causality. Likewise, the same is true for bugtracker entry updates *B* (comments) added at time *t*_*u*_(*B*) that appear to reference commits *C* made only at a later time *t*(*C*).

There are three exceptions to these rules: (1) Git has a feature called “rebase” that allows to create time-travel effects at will by removing a commit from the history and reinserting an equivalent one at any other point into the history. In practice, this is used for eliminating branches and the reinsertion will be at a later time, not an earlier one. Also, the commit changes its ID. So this is not a problem. (2) Git allows changing the content or timestamp of a commit message later. This feature is not normally used at Infopark, so this is not a problem either. (3) If the time difference is only small, it might be due to a misalignment between the bugtracker server clock and the version archive server clock. We therefore accept references into the very near future (a few minutes), but not into a farther future, as those could only be valid in the presence of lucky guessing or time travel—and history teaches us that both of these are not common in software development.

*Heuristic TT.* (*C*) For bugfix links established by a commit message *C*, consider them invalid if *t*(*C*) + *t*_*m*_ < *t*_*c*_(*B*). (*B*) For bugfix links established by a Bugzilla entry *B*, consider them invalid if *t*_*u*_(*B*) + *t*_*m*_ < *t*(*C*). Choose a suitable clock misalignment tolerance *t*_*m*_.

### 5.7. Filter LU: Reject Late Updates of Bugzilla Entries

If a commit *C* is referenced from a Bugtracker entry *B* at all, we would expect that reference to appear shortly after the commit: usually only minutes later, possibly hours, or potentially the next day, but probably not later than that because the developer would simply forget to add it. This is true for both previously existing Bugtracker entries (the normal case) as well as entries created post hoc (after the commit), in which case the reference might even appear in the initial bug description rather than a subsequently added comment.

*Heuristic LU.* (*B*) For bugfix links established by a Bugzilla entry *B*, consider them invalid if *t*_*u*_(*B*) > *t*(*C*) + *t*_*w*_. Choose a suitable maximum wait time *t*_*w*_.

### 5.8. Filter UD: Reject All Merely Unidirectional Bugfix Links

Any single bugfix link may be spurious and the above heuristics attempt to identify it as such in a context-free manner, without looking at any other link. However, as there are tens of thousands of commits and thousands of bugtracker entries, it is unlikely that a spurious bugfix link from, for example, commit *C* to bugtracker entry *B*, has an also spurious counterpart in *B* accidentally pointing to *C*. If we* require* both of these links to exist, we can expect them to be valid.

This is a potentially ruinous filter. Unless it is common in the development organization to mention commit IDs in bugtracker comments, the rule will be far too strict and will result in extremely low recall. In the Infopark case, however, it turns out to be practical and should be considered because it promises high filtering precision. Also, this filter does not require a tuning parameter to be chosen.

## 6. Tuning, Results, and Discussion

This section describes how we select the tuning parameters of the filters and what performance is thus obtained with each. The methods for choosing each tuning parameter do not use the ground-truth knowledge from our manual assessment of the candidates, but rather are procedures as they could be applied by any engineer attempting to perform a good search for bugfix links automatically. These procedures are based on simple diagnostic plots and invoke human judgment, so that relevant background knowledge is not thrown away if the engineer has such knowledge. We will describe this reasoning for each filter.

In each case, we will describe three choices of parameter: (1) a* loose* choice, which emphasizes filtering precision so as to minimize results loss; (2) an* aggressive* choice, which emphasizes strength as long as filtering precision is still over 50%; If successful this should result in a larger improvement of overall precision and also in higher results loss; if unsuccessful, the filter might ruin recall* and* hurt overall precision at the same time; (3) a* default* choice, which trades off these two risks.

Section 6.9 discusses how the filters should be combined and what performance is thus achieved overall.

### 6.1. Generator BM: Bugtracker IDs Mentioned in Commit Messages

Generator BM suggested 4037 candidate bugfix links. These have a precision of 79%.

### 6.2. Generator CM: Commit IDs Mentioned in Bugtracker Entries

Generator CM suggested 3015 candidate bugfix links. These have a precision of 60%.

Both generators combined suggested 5005 candidate bugfix links (of which 2047 are bidirectional). These 5005 have a precision of 73%, the starting point of our filters' precision improvement work.

*Recall.* The mean answers in our survey (as described in Section 4.5) suggested 35.8% of all commits to be bugfix commits (with standard error 15.1%). But no better one is available and 71% of those to have a corresponding Bugzilla entry. Our data covers 19 955 commits. If we assume that the survey answers reflect the same notion of bugfix as our data, at 100% recall the data* should* provide 5 072 unique bugfix link candidates. It* does* provide 5 005, which have a pessimistically estimated precision of 73%, so there are at least 3653 correct ones and the estimated initial recall is thus estimated as at least 72%.

We emphasize that this estimate is very rough. It has an unknown and possibly large margin of error. Its only purpose for Infopark was to make the decision whether the required “at least 50% recall” (see Section 3.1) could likely be met. This decision was positive at this point. Subsequent filtering produces results loss and so will diminish recall; see Section 6.9 for the eventual final value of roughly estimated recall.

### 6.3. Filter FB: Reject Overly Frequent Bugzilla IDs

**Figure 1:**
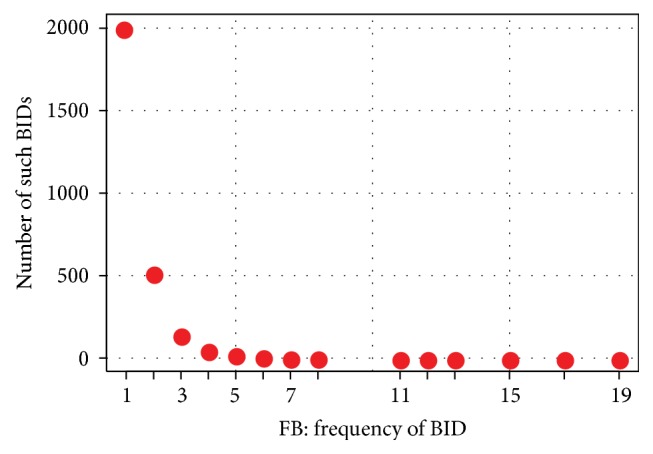
How many Bugzilla IDs (*y*-axis) are referenced exactly *x* times.

For choosing *f*_*B*_, that is, how often a Bugzilla ID must occur to be filtered out, we plot in Figure 1 how often each referencing frequency occurs and reason that only rare amounts of repetition can safely be filtered out. The plot suggests 5 as the loose choice (looking to lie barely above the zero line), 3 as the aggressive choice, and we pick the reasonably safe-looking 4 as default choice.

It turns out that 3 is in fact overly aggressive and results in a filtering precision of only 39% and a hurtful results loss of 12%. 4 (and 5) works alright at filtering precision 50% (60%) and resulting results loss of 6% (3%). All three parameters nevertheless result in the same overall precision of 77%, so the loose choice would have been clearly best in this case.

For a complete overview of the performance statistics for the default parameter choice of all the filters, please refer to Table 1.

### 6.4. Filter FC: Reject Overly Frequent Commit IDs

**Figure 2:**
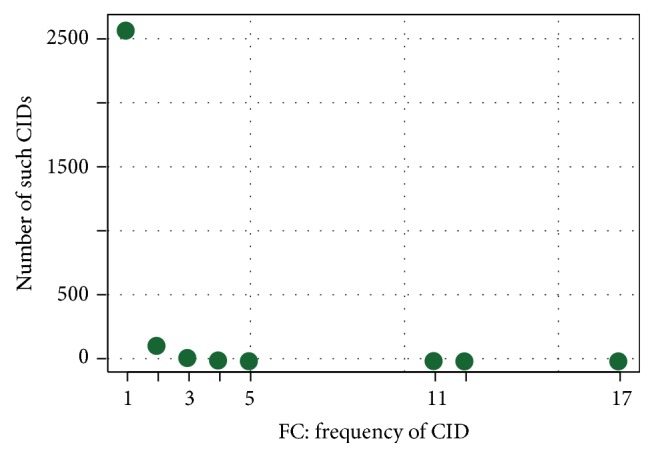
How many commit IDs (*y*-axis) are referenced exactly *x* times.

Analogously, for choosing *f*_*C*_, that is, how often a commit ID must occur to be filtered out, we plot in Figure 2 how often each referencing frequency occurs. Considering that candidate commit IDs found are very likely to really be commit IDs (as opposed to candidate Bugzilla IDs which are just integers) we decide to tread a lot more carefully here and to* not* consider 2 as the aggressive choice. We select 3 as the aggressive and default choice and 4 as the loose choice.

It turns out that this filter is very precise. Filtering precision for 3 (4) is 96% (99%). However, as the strength is only 4% (2%), the impact of the filter is modest, with results losses below 0.3% and overall precision of 77% (76%).

### 6.5. Filter SB: Reject Small Bugzilla IDs

**Figure 3:**
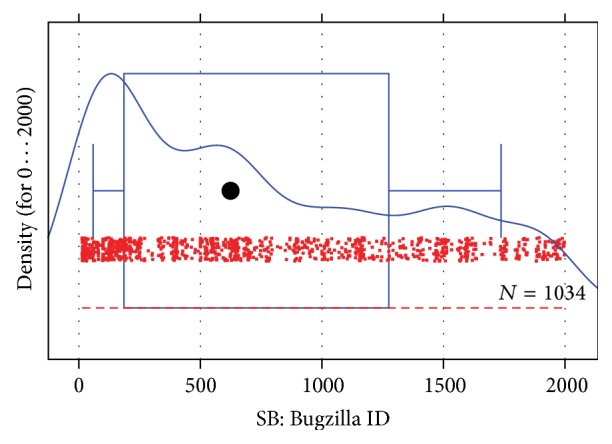
Density of the occurrence of small and medium small candidate Bugzilla IDs. The boxplot shows percentiles 10, 25, 50, 75, and 90. The density estimator was computed by the density function of the R statistical software system (version 2.12) using default parameters.

We plot the density of Bugzilla IDs. For readability we restrict the plot to maximum 2000 (this covers the bottom 26% of all IDs) and obtain the plot shown in Figure 3. There is no obvious cutoff point, but several candidates. The first would be the peak of the distribution at about 130, then the first turning point around 350, the second peak at around 600, and the second turning point around 750.

We choose 130 as the loose choice and 750 as the aggressive choice. Considering that the raw data values look like a good mix of quite a few different values (rather than just a very few values occurring over and over), we decide not to be too aggressive and pick 350 as default choice. The mix of many different values also means the filter will not overlap too strongly with FB.

These considerations are all quite valid and successful. Filtering precision of the loose/default/aggressive choice is 92%/81%/56% and the resulting overall precision is 78%/81%/81%, so being aggressive is not helpful in this case. Results loss is 0.3%/1.3%/5%.

### 6.6. Filter TT: Reject Major Time Travel

**Figure 4:**
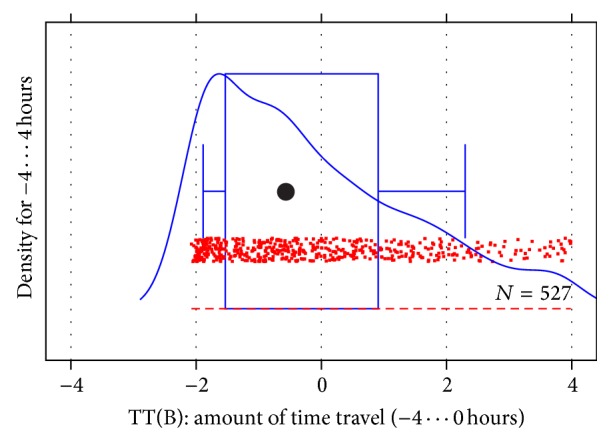
Time difference from creation of Bugzilla entry to time of commit that mentions it.

We expect that the time travel filter to work perfectly once we account for a few minutes of clock drift. However, the two diagnostic plots used each hold a surprise.  Figure 4 reveals massive occurrence of time travel of about 1 hour and even more of about 2 hours. This indicates a time zone error on the servers. Infopark is in time zone GMT+1 in winter and GMT+2 during daylight saving time. Apparently the version archive runs on correct time but the Bugzilla server's clock is one or two hours in the future (e.g., runs on local time but claims it to be GMT). So when a Bugzilla entry is created it will be recorded as two hours later than true so that a fast subsequent commit mentioning its ID appears in the past. As for actual time drift, the minimum value is 2.08 hours, indicating 5 minutes maximum drift. We choose 2.1 hours as our loose, default, and aggressive parameter value for the clock misalignment *t*_*m*_ for TT(B).

**Figure 5:**
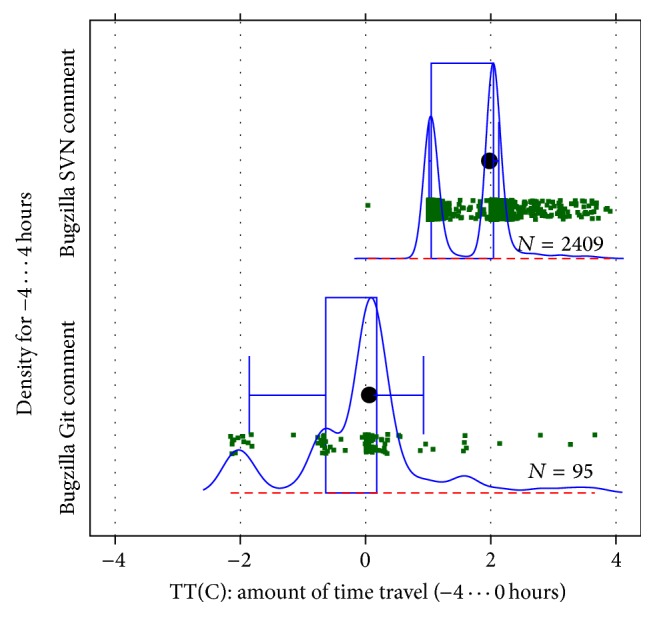
Time difference from commit to time of Bugzilla comment that mentions it.

Figure 5 reveals something else to be odd in our data. The plot shows that the time until a commit is mentioned in a Bugzilla comment. It should indicate the opposite effect. If the time zone problem was present throughout the lifetime of our data, nobody should (appear to) be able to mention a commit faster than after 1 hour (or 2 hours, resp.). The SVN part of our data shows just this behavior. In contrast, the (much smaller) Git part is mostly correct. However, a fraction of it exhibits time travel in the opposite direction, as if the Bugzilla and version archive servers had now changed roles, which does not sound likely indeed. We were not able to find out the origin of this inconsistency in our data so we did what a practicing engineer would do (given the small number of data points affected): live with it as it is. We hence apply the same *t*_*m*_ of 2.1 hours for TT(C) as well.

Both TT(B) and TT(C) are very weak, with a strength below 1% and almost no results loss at all. TT(C) has filtering precision 43% and overall precision 73%. TT(B) has filtering precision 99% and overall precision 74%.

### 6.7. Filter LU: Reject Late Updates of Bugzilla Entries

**Figure 6:**
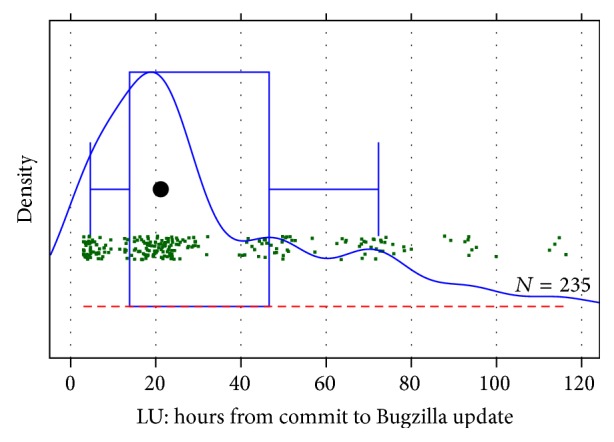
Time from commit to the appearance of its mention (if any) in a Bugzilla entry.

We use the same plot as for TT(C) but now we focus on the positive time range. We add 1.5 hours to minimize the distortion from the time zone problem. The density plot is not useful if we start the plot at time zero, as by far the most activity is early, not late, so we start the density estimation only after 4 hours as shown in Figure 6.

As Infopark is a one-time zone company, the plausible cutoff points are in the nights. The first after 10 hours (clearly overly aggressive; not visible in the density plot because of our removal of the first 4 hours), then after about 32 hours, 55 hours, 80 hours, and 100 hours. An informal survey among Infopark developers suggested “three days” as a good cutoff, so we pick 32 hours as the aggressive choice and 80 hours as the default and loose choice.

The difference is not large: filtering precision for 32 (80) hours is 56% (62%), results loss is 3% (2%), and overall precision is 77% in both cases.

### 6.8. Filter UD: Reject All Merely Uni-Directional Bugfix Links

This link uses a qualitative criterion and does not have a tuning parameter.

Since the majority of bugfix links is unidirectional, this filter's strength is high (59%); its filtering precision is low: only 29%. The results are rather extreme. Results loss is an unacceptable 42%, but the resulting precision is a brilliant 99%.

### 6.9. Filter COMBI: Optimized Overall Filtering

Given these properties of filter UD, it is obviously not helpful to combine it with the others in a successive filter chain, as even UD alone is too aggressive and has too much results loss.

So we turn UD around and use it as an* acceptance* criterion instead, Accept a candidate if it passes all of the remaining filters FB, FC, SB, TT(B), TT(C), and LU, but also accept it if it is bidirectional. We call this filter COMBI.

In the loose parameter choice for each filter, the resulting COMBI filter has filter precision 70% and a strength of 10%. It achieves an overall precision of 88% at 4.1% results loss; a very good result. The default filter is even better: its filter precision of 63% leads to 93% overall precision at just 7.1% results loss. The aggressive version is a little less efficient: with filter precision 48% it achieves 94% precision, but at the cost of 14% results loss (strength 27%).

Table 1 shows the effect of each filter (with default parameter) invididually and the effect of chaining them in order of increasing strength and then applying UD acceptance as the last step. As we see, all filters make a positive contribution to overall precision. The first three (TT(C), TT(B), and FC) raise overall precision from 73% to 78%, yet produce only negligible results loss. The next three, (LU, SB, and FB) increase overall precision impressively from 78% to 93% but also pile up some results loss of 9.3%. The subsequent UD-driven acceptance stage leaves precision essentially unchanged but cuts the results loss down to 7.1%.

For the loose parameter choices, the acceptance stage reduces results loss from 5.3% to 4.1%; for the aggressive choices it is from 19% to 14%. Overall, this is a smooth and effective ensemble of filters.

As for final overall recall, we need to go back to the estimate of initial recall in Section 6.2. Results loss of 7.1% on 5005 candidate links means we lost 355 valid links. The generators produced an estimated 3653 valid links at least, or at least 72% initial recall, so now we end up with at least 3298 valid links or at least 65% final recall. Again, please keep in mind that this estimate is very rough but the reduced value still sufficed for Infopark to decide they were meeting their own requirement of “at least 50% recall” put up in Section 3.1.

## 7. Bflinks: How to Use the Method in Practice?

For clarification, we will now summarize how one would apply the overall bflinks method to one's own repository and how one can copy with the potentially large variation in repository content and properties that may occur due to different application domain, technology used, development conventions, and idiosyncrasies.

The procedure has to be applied by somebody who knows and understands the repository content well, in particular the text of the commit messages and bugtracker descriptions and comments.

1. Make the version archive and bugtracker data accessible and run the generators BM and, if applicable, CM.
2. Create the diagnostic plots for choosing the cutoff parameter for each filter as described in Sections 6.3 to 6.7.
3. Based on each plot, combined with your understanding of repository content, select a loose, default, and aggressive parameter setting for each filter. Expect to find potentially much-different values compared to those in the present paper. For instance, your bugtracking procedures may produce much higher numbers of correct mentions of some bug IDs (affects filter FB), your quality assurance procedures may produce much later correct updates of bugtracker entries (affects LU), your application domain may involve other number ranges of non-ID numbers mentioned in commits (affects SB), your server clocks may have less or more time and time zone issues (affects TT), and so on.
4. Using the default parameter, compute filter strength for each filter. If those are similar to the strengths reported in this paper or conform to your repository-specific expectations for some other reason, you may be willing to trust the values and hence the filters and start using the filter chain in this form.
5. Otherwise, switch to the loose or aggressive setting for the problematic filters.
6. Sort the filters by increasing filter strength and compute overall filter strength along the chain as shown in the right half of Table 1. If those filter strengths are similar to the strengths reported in this paper or conform to your repository-specific expectations for some other reason, you may be willing to trust the values and hence the filters and start using the filter chain in this form.
7. Otherwise, draw a random sample of 100 candidate links, apply the filter chain to them, and manually validate the results. Compute precision. Compute results loss. If necessary, adjust the parameter setting of your strongest two or three filters until you obtain a reasonable tradeoff between precision and results loss.
8. If results loss appears unacceptably high, your repository is not well suited for bugfix link identification.
9. If precision appears unacceptably low, add syntax matching and keyword matching to the BM generator so that it does not use every integer found but instead requires forms such as  # 1234 (or whatever is commonly used in your organization) and/or requires keywords such as bug, fix, and fixes—this is not described above. Remove the SB filter in this case.
10. If precision and results loss are now acceptable for the sample, this provides you with a rough estimate of true precision and true results loss with approximately the following precision. According to the binominal distribution, for observed frequencies of false positives or false negatives of 5% (or 10% or 25%), with a probability of 90%, the actual value will be in the range 2%–9% (or 5%–15% or 18%–32%, resp.).

Keep in mind that no amount of quality of the method (or validation of that quality for other repositories) and no amount of understanding of your particular repository content on your side will make a successful bugfix link identification happen if the repository content has too few correct and/or too many misleading mentions of IDs. See also the discussion of external validity below.

## 8. Threats to Validity

### 8.1. External Validity

It should be clear from the above discussion that the results of bugfix link filtering strongly depend on the development practices of the organization.

Our results serve to explain certain methods by which good results can be obtained, but the actual results are clearly specific to our particular case and might be very different elsewhere.

In particular, the results could be better if the discipline of mentioning Bugzilla IDs and commit IDs were higher or far worse if it were lower; they could also be better if a fixed syntax for Bugzilla ID mentions were used throughout; they could be worse if more Bugzilla IDs or commit IDs were mentioned in other roles than the bugfix link role.

One particularly important issue for the generalizability of our method and findings is the frequency of backward links from the bugtracker to a particular commit. If this were a common practice in the large old version repositories of popular projects such as Apache and Eclipse, somebody would have invented the use of backward links long ago. But how about more recent environments? In particular, the Git version management system (http://git-scm.com/, first released in 2005.), suggests (via its “cherry-picking” functionality) very fine-grained commits* in order to describe the semantics of each change discriminately*.

To determine whether such technology leads to a development culture with many backward links, we performed a study on GitHub (http://github.com, founded 2009.) as follows.

- We searched for the super-generic keyword “project,” which gave 151730 project hits (repository hits) in apparently random order.
- We took the first 100 of these projects and selected all those that had at least 1000 followers (stars), which resulted in 16 projects ranging from 1041 to 7990 followers.
- 3 of these projects did not use the issue tracker at all.
- For each of the others, if they used tags, we reviewed the 30 youngest closed tracker entries tagged “bug,” “confirmed,” or “defect.”
- For projects not using tags, we reviewed all “issue” entries among the 30 youngest closed entries and excluded only those that were very obviously not about a bug (but rather about a feature request or configuration issue).
- Of these tracker entries, between 0% and 100% (per project) provided backward links to a commit, only one project was below 20%. The average over the 13 projects was 44%, which happens to be quite close to our own data (41%).

We conclude that the applicability of the powerful UD filter will likely be good at least in many younger projects using Git and GitHub.

### 8.2. Internal Validity

The manual validation of bugfix links is boring, quasirepetitive work and is likely to contain some amount of error even for the repository expert. We estimate this error to be on the order of 1% to 2%—too little to modify our conclusions.

Since we have validated only a subset of all bugfix links, the precision measurements also involve sampling error. As described in Section 4.5, this error is biased and distorts our results towards lower-than-actual precision results, so that the actual performance of the method was higher than we have reported. Due to the large size of the validated sample, the difference will be small, though.

As mentioned in Section 6.2, our recall estimate is very unstable. A margin of error of about ±20 percentage points should be assumed here. However, we do not consider a particular value of recall to be part of our results.

## 9. Conclusions

In this paper, we have described how to use a chain of different filters on candidate bugfix links in order to obtain high precision without loosing much recall. In particular, we have shown how to tune those filters' cutoff parameters and how to make use of backward links from defect database to version archive if such links are available. We have evaluated the techniques on a rather large commercial repository by carefully and individually checking over 2500 candidate bugfix links. There are a number of conclusions as follows.

1. The ambitious quality criteria set by Infopark for avoiding incorrect conclusions (at least 50% recall and at least 80% precision) were met for the bugfix links. Our generator/filter network achieved roughly 65% recall (please observe the discussion in Sections 4.3, 6.2, 6.9, and 8.2.) and at least 93% precision. Remember, however, that a lot of headroom will be required for the subsequent bug insertion localization step, so this is not as big a success as it may seem.
2. We present 7 filters, 6 of which have a cutoff parameter that needs tuning. The simple diagnostic plots and tactical reasoning we describe to be used for parameter tuning have worked very well. In all 6 cases the results were good, in 5 of the 6 cases they also had the properties that were expected; only the FB filter parameter choice turned out to be more aggressive than expected and needlessly aggressive, too. These approaches and their general idea are practical and appear to be helpful and sufficiently safe. While we have demonstrated a practical approach to choosing the cutoff values with the help of diagnostic plots, the particular parameter tuning decisions made with this approach need to be idiosyncratic and must take specific properties of the product and the development process into account.
3. After tuning, it took 5 of the 6 filters to be chained to cross the precision threshold of 80%. We conclude that filtering must not be done too timidly and many filtering ideas (possibly organization-specific ones) should be combined.
4. Results loss can be used to guide the aggressiveness in parameter choice by means of some manual classification of filtering results where needed.
5. Unconditional acceptance of bidirectional links helps limiting results loss. Much less results loss needs to be accepted in order to achieve the same high precision if (and only if) a high density of backward links from bugtracker entries to commits is available.
6. Thus, we recommend regularly mentioning bugfix commit IDs in bugtracker comments as a development practice.

## Figures and Tables

**Table 1:** Performance of each successive stage of the default COMBI filter: filtering precision (*fp*), filter strength (*st*), results loss (*rl*), and overall precision (*op*) achieved by this filter alone or together with all preceding filters. All entries are in percent.

	This one filter solo				Filters up to here together			
	*fp*	*st*	*rl*	*op*	*fp*	*st*	*rl*	*op*
TT(C)	43	0.3	0.2	73	43	0.3	0.2	73
TT(B)	99	0.9	0	74	86	1.2	0.2	74
FC	96	3.7	0.2	77	93	4.9	0.4	78
LU	62	5.6	2.1	77	74	8.3	2.7	79
SB	81	6.9	1.3	81	67	15	4.0	90
FB	50	11	5.6	77	60	23	9.3	93
UD^a^	29	59	42	99	63	19	7.1	93

^a^“Filters up to here together” uses UD in accepting mode, not rejecting mode.
